# Comparison of heart rate response and heart rate recovery after step test among smoker and non-smoker athletes

**DOI:** 10.4314/ahs.v21i1.15

**Published:** 2021-03

**Authors:** Garyfallia Pepera, Zogka Panagiota

**Affiliations:** Clinical Exercise Physiology and Rehabilitation Laboratory, Department of Physiotherapy, University of Thessaly, 3rd km of Old National Road, GR-35100 Lamia, Greece

**Keywords:** Smoking, athletes, heart rate recovery, heart rate reserve, six-minute step test

## Abstract

**Background:**

Exercise performance depend on the ability of the cardiovascular system to respond to a wide range of metabolic demands and physical exertion.

**Objectives:**

To investigate the habitual smoking effects in heart rate response and heart rate recovery after step test in athletes.

**Methods:**

Seventy-eight physically healthy active athletes (45 non-smokers and 33 smokers) aging 27±8 years old, participated in this study. All participants completed the International Physical Activity Questionnaire and performed the six-minute step test. Cardiovascular parameters such (resting heart rate, peak heart rate, heart rate at 1 min after testing, heart rate recovery, recovery time, blood pressure at rest, and post-testing blood pressure) were recorded.

**Results:**

Smoker-athletes had higher resting heart rate (76 ± 9bpm vs. 72 ± 10bpm, p<0.05), maximum heart rate (154 ± 18bpm vs. 147 ± 17bpm, p<0.05) and recovery time (7min 25sec ± 6min 31sec vs. 4min 21sec ± 4min 30sec, p<0.05) than non-smoker athletes. Scores from the IPAQ were approximately the same (M=7927 ± 10303, M= 6380 ± 4539, p<0.05).

**Conclusion:**

Smoking was found to affect athletes' cardiovascular fitness. The change of the athletes' heart rate recovery and recovery time contributes to the adaptation of cardiovascular function in training requirements.

## Introduction

Smoking-related diseases are one of the most important causes of premature death globally.^1^ According to the European Society of Cardiology, smoking provokes 28% of cardiovascular deaths in men and 13% in women, aged 35 to 69 years.^2^ Prolonged smoking has many effects on the cardiovascular, respiratory and circulatory system, increasing the incidence of coronary artery disease and myocardial infarction.^3^

Additionally, is implicated in autonomic dysfunction^4^ and increases myocardial workload. The high-intensity aerobic training significantly reduced resting heart rate and induced positive changes in resting autonomic modulation by parasympathetic activation and sympathetic withdrawal in apparently healthy male smokers.^5^ Smokers had a higher or same resting heart rate, maximum heart rate, and heart rate during exercise compared to non-smokers.^6^–^11^ There is a non-invasive and easy-to-measure index of myocardial work and consequently cardiovascular health.^12^

Exercise is the most pronounced condition to submit a normal cardiovascular system. Exercise is associated with various of cardiovascular adaptations, such as an increase in heart rate, maximal oxygen uptake, and blood pressure as a response to increased oxygen demand.^11^ Long-term cardiac adaptations to exercise, includes reduction in resting heart rate and heart rate recovery.^1^,^7^ Heart rate recovery (HRR) is an index of physical fitness and cardiovascular risk predictable.^13^ HRR is defined as the difference between peak heart rate and 1 minute into the recovery time after exercise or testing.^13^,^14^ HRR measurement is widely used in clinical practice as one of the inexpensive, valid and the most important simple indicators of the autonomic nervous system activity.^15^

There are a large number of athletes in the world who smoke, as smoking is the most common bad habit of athletes.^16^ The present study aimed to determine the interaction of smoking through the exercise in cardiovascular function and physical fitness. Furthermore, the cardiovascular health and physical activity are determined in miscellaneous sports. It tested the hypothesis that heart rate responses to cigarette smoking is implicated in the link between smoking and poor cardiovascular function.

## Materials and methods

The sample consisted of 78 amateur athletes, among whom 33 were smokers and 45 were non-smokers. Table 1 summarises the clinical characteristics and baseline values of the sample. Athletes were recruited via sports clubs across Greece. Exclusion criteria included pre-existing cardiovascular conditions and comorbidities (e.g. diabetes, hypercholesterolemia, hypertension). In addition to the above, athletes who had resting heart rate greater than 120 beats per minute (HR rest > 120bpm) or systolic blood pressure greater than 180 mmHg (SBP rest > 180mmHg) or diastolic blood pressure greater than 120 mm Hg (DBP rest > 120mmHg) in the rest conditions, were excluded from the test and were not further evaluated. Each participant gave written informed consent, after receiving an information sheet outlining an explanation of the procedure. All procedures were approved by The University Ethics Committee and conformed to the declaration of Helsinki guidelines for research with human participants.

**Table 1:** Descriptive analysis of sample

	Smokers (N= 33)	Non smokers (N= 45)
**Age (yrs)**	27 ± 7	27 ± 8
**Weight (kg)**	71.54 ± 9.73	79.60 ± 14.01
**Height (m)**	1.74 ± 0.07	1.78 ± 0.1
**Amount of smoking (cigarettes)**	11.45 ± 8.49	0
**Sports:**		
**Tennis**	1	3
**Brazilian JiuJitsu**	4	5
**Kick Boxing**	8	11
**Kung Fu**	2	1
**Basketball**	4	13
**Football**	14	10
**Weightlifting**	-	1
**Running**	-	1

### Study design

Before testing, each participant received a primary health assessment (pre-exercise health questionnaire) and interview (medical, pharmacological, and family history).

### International physical activity questionnaire short

Physical activity was assessed via the short version of the International Physical Activity Questionnaire Greek Version. The above questionnaire concerns the time they spent being physically active in the last seven days. Based on the IPAQ scoring procedure, physical activity status was classified into three categories: low physical activity (total PAscore< 600 MET.min.wk-1), moderate physical activity ≥ 600 MET.min.wk-1 and high physical (total PAscore ≥ 3000 MET.min.wk-1 or vigorous PAscore≥ 1500 MET.min.wk-1). 17

### Six-minute step test

The six-minute step test was performed to evaluate the exercise capacity. Participants performed a six-minute step test by step onto a 17cm tall wooden step at a rate of 40 steps per minute. Participants were refrained from smoking, drinking, eating, and strenuous exercise for at least three hours before the test. Heart rate was recorded at the beginning of the test, at the end of 1^st^, 2^nd^, 6^th^ minute, at the end of the test, 1-min after the end of the test. Heart rate recovery (HRR) was calculated as the difference between maximal exercise heart rate and HR 1-min after test termination. Total heart rate recovery time was also recorded as the time to resting Heart rate ± 10 bpm. Blood pressure was measured at the end of each test.

Blood pressure (BP) was measured before testing (resting BP) and within one minute of test termination (post-exercise BP), using an automated arm blood pressure machine (Omron Digital Automatic Blood Pressure Monitor MX3 Plus, Omron Health Care CO., Ltd).

### Statistical analysis

Statistical analysis was implemented using statistical package for the Social Sciences (SPSS Chicago, IL, USA). Independent samples t-test used to compare the dependents variables (resting heart rate, maximum heart rate, heart rate recovery, heart rate in the first, second, and sixth minute, recovery time) among the smokers and non-smokers, with a significance level of 95% (p<0.05). Mann-Whitney test for IPAQ score, continuous non-parametric data, was used to examine differences between the smokers and non-smokers, with a significance level of 95% (p<0.05). One-way ANOVA test was used to compare the cardiovascular parameters among six types of sport (kung fu, kickboxing, football, basketball, tennis, and Brazilian jiu-jitsu). Finally, the effect size was calculated by Cohen's d.

## Results

### Anthropometric parameters

No significant relationships were demonstrated between several independent variables such as age, weight, height, and dependent variables, such as resting heart rate, maximum heart rate, heart rate recovery, and recovery time (Table 1). The amount of smoking consumed from three to forty cigarettes, daily. Females hadon average a resting heart rate 3bpm higher (76 ± 10 bpm vs 73 ± 10 bpm), a maximum heart rate 6 bpm lower (M= 144 ± 17 bpmvs M= 151 ± 18 bpm) and a heart rate recovery 3 bpm higher (M= 48 ± 17 bpm vs 45 ± 12 bpm) than men.

### Cardiovascular parameters

The variance analysis using the Independent Samples t-tests showed that across all cardiovascular parameters (HRresting, HRmax, HRrecovery1, recovery time) there were statistical significant differences between smokers and non-smokers athletes (p<0.05). Table 2 cites the effects of smoking on cardiovascular parameters. Smokers had significantly 4 bpm higher resting HR than non-smokers athletes (p<0.005). The maximum heart rate achieved (HRmax) was significantly 7 bpm higher for smokers compared to non-smokers athletes (p<0.005). The HR during recovery 1 min after maximum exercise was significantly lower in the non-smoker athletes compared to the smoker athletes (p= 0.04). Moreover, heart rate 1-min after test termination was a 12 bpm higher in smokers compared to non-smokers athletes (p<0.005). Total heart rate recovery time was twice higher in smokers athletes than non-smokers. Smokers athletes had a 5 bpm significantly lower total heart rate recovery in comparison with the non-smokers (p < 0.005). The effect size of resting heart rate, maximum heart rate and hearte rate 1-min after the termination was 0.15, 0.19 and 0.29, respectively.

**Table 2:** Variance analysis of the effects of smoking on heart rate values at rest, during sub-maximal exercise, at peak exercise and during recovery

	Smokers *(n=33)*		Non- Smokers *(n=45)*		Mean difference 95% CI	t-test	Sig.(2-tailed) p<0.05
	
	Mean	(SD)	Mean	(SD)			
**HRrest (bpm)**	76	(9)	72	(10)	3.50 (-85.2 to -70.9)	-22.3	<0.005
**HRmax(bpm)**	154	(18)	147	(17)	2.28 (-78.7 to -69.5)	-32.4	<0.005
**HR1minμετά (bpm)**	112	(19)	100	(18)	4.91 (59.2 to 79.2)	32	<0.005
**HRrec. (bpm)**	42	(13)	47	(12)	3.89 (45 to 60.7)	44	<0.005
**Recovery** **time (min sec)**	7m 25s	6m 31s	4m 21s	4m 30s	74 (4s to 5m 8s)	2.1	0.04

*HR1min – Heart rate in the first minute after the end of exercise.

### International Physical Activity Questionnaire (IPAQ)

The Mann-Whitney test findings were presented as median and range values and were showed the association between IPAQ score and smoking (Table 3). Three participants excluded because they were unable to answer the questions with certainty. IPAQ score was approached the boarderline of significance between smokers and non-smokers athletes (median= 6900, range= 47280 vs median= 4380, range= 41280, p= 0.08).

**Table 3:** Median and range values of IPAQ score in smokers and non- smokers, athletes (Mann-Whitney test)

	Non smoker *(n=43)*	Smoker *(n= 32)*	P value
**IPAQ score**	4380 (41280)	6900 (47280)	
**Mann- Whitney test** **Asymptotic Sig.(2-sided test)**			0.08

### Classification of sports

One way Anova test was used to correlate the cardiovascular function with sports (kick boxing, football, basketball, Brazilian jiu-jitsu, tennis, kung fu). According to the findings of the test, the recovery time (p=0.008) and the maximum heart rate (p=0.0016) used to the classification in miscellaneous sports (Table 4). Basketball players (22.8%) had the lowest heart rate recovery time (M= 2min 45sec, SD= 2min 16sec) of all participants in sports. Tennis was the second sport in the classification, because the tennis players (5.1%) need more time to recovery (M= 3min 8 Sec, SD=1min 45 sec). Athletes from Kung Fu (3.8%) had lower time for recovery (M= 3min 42 sec, SD= 2min 13sec) from the football players(30.8%)(M= 5min 11sec, SD= 3min 33sec), who had more recovery time than tennis and basketball players. At the end of classification with the most time by all were the athletes from Kick Boxing (23.3%) (M= 7min 12sec, SD= 6min 26 sec) and Brazilian JiuJitsu (11.5%) (M= 11min 32sec, SD= 9min 31sec).

**Table 4:** Mean and SD values of recovery time and maximum heart rate in miscellaneous sports

Sport	Recovery time *(p<0.05)*	HRmax*(p<0.05)*
**Basketball**	2min 45sec ± 2min 16sec	145 ± 12 bpm
**Tennis**	3min 8sec ± 1min 45sec	138 ± 11 bpm
**Kung Fu**	3min 42sec ± 2min 13sec	163 ± 3 bpm
**Football**	5min 11sec ± 3min 33sec	148 ± 13 bpm
**Kick Boxing**	7min 12sec ± 6min 26sec)	150 ± 21bpm
**Brazilian JiuJitsu**	11min 32sec ± 9min 31sec	166 ± 22 bpm

Athletes' HRmax values were lower than those who were not regularly exercised.^7^ Tennis players had lower HRmax(M = 138 ± 11 bpm) than other athletes (basketball players: M = 145 ± 12 bpm; football players: M = 148 ± 13 bpm and kick boxing players: M = 150 ± 21 bpm). However, Kung Fu and Brazilian JiuJitsu athletes had greater maximum heart rate values, M= 163 ± 3 bpm and M= 166 ± 22 bpm, respectively.

## Discussion

This is the first prospective study that examined the effect of cardiovascular, anthropometric parameters, and smoking on a six-minute step test among young, athletes. The findings of the present study showed that the mean of smokers' resting heart rate was 4 bpm greater than non-smokers. Also, the maximum heart rate was 7 bpm greater in young, athletes, smokers. Eratet al^10^, similarly reported that there is a difference in resting heart rate and maximum heart rate.

Heart rate recovery is considered an important cardiovascular prognostic index.^13^,^18^,^19^ Smokers had significant (p<0.05) 5bpm higher heart rate recovery than non-smokers. Also, the results of Erat et al^10^ study showed that the heart rate recovery at the first minute after the exercise test in rest was higher in smokers by 6bpm than non-smokers. Sydo et al (2018)^11^ reported that there was a significant difference in heart rate peak between smokers and non-smoker group, by 7 bpm. Also, the heart rate recovery, which defined as peak exercise HR minus HR at 1 minute of active recovery was observed greater in non-smoker group. The study of Jin-Jang et al (2017)^20^, comparison of smoking on cardiopulmonary function in taekwondo athletes showed a similar tendency of the results in this study, despite the different definitions of heart rate recovery.

On the other hand, Seller et al (2009)^6^ and Hannifah et al (2013)^8^, found that there were no significant differences in heart rate recovery between the groups, because the sample contained an unequal number of smokers and non-smokers.

As reported by the research of Papathanasiou et al^21^ study showed that there was significantly lower maximum heart rate in smokers than non- smokers, but this phenomenon was only seen in the women. However, a higher and faster decrease was observed in the heart rate at the first minute after the exercise test, both in females and males non-smoking group.

Furthermore, low abatement of recovery time value is an important factor in cardiovascular function and fitness.^22^ Smokers' recovery time (7min 25sec ± 6min 31sec) to the resting level was double than non-smokers (4min 21sec ± 4min 30sec). Kabayashi et al^9^ study showed an increased recovery time in healthy smokers, although the recovery time had a different definition.

Another important finding of the present study was the classification of physical fitness in different types of sport which was created by the mean of the heart rate recovery time. The sports ranked by lower heart rate recovery were basketball, tennis, kung fu, football, kickboxing and Brazilian jiu-jitsu. The above is justified by the fact that smoking reducts cardiorespiratory fitness in the lower body exercises, whereas there are no effects on the upper body exercises^23^. So, athletes who used lower limb exercise in their sport (kickboxing, Brazilian jiu-jitsu, football) had the higher negative effect of smoking caused significant reductions in cardiorespiratory fitness. Also, Gharbiet al (2015)^24^ recorded VO_2max_, index of cardiopulmonary resistance^6^, in sixteen athletes and demonstrated that basketball players had greater VO_2max_ than football and handballplayers.As a result, the basketball players had better cardiorespiratory function than football and handball players.

In contrast to earlier findings, no evidence of the association of the level of physical activity among smokers and non-smokers was detected (M=7927 ± 10303, M= 6380 ± 4539, p= 0.06). It may be the case that the sample of this research had consisted of active athletes who were training daily for the best possible performance in sport. Daily activities and regular workout are factors that should have been taken into account in the present results. Papathanasiou et al^21^ has been proven that there was no statistically significant correlation between the sample of healthy adult students and the level of physical activity, as it was the measurement by the IPAQ questionnaire.

Contrary to expectations, this research did not find a significant difference between the anthropometric variables and cardiovascular function and fitness. Age, height, weight, and gender were independent of physical fitness and cardiovascular function. Other studies^6^,^8^, ^10^,^11^,^25^–^29^ are confirmed that the anthropometric measurements play a significant role in influencing physical fitness and cardiovascular parameters. On the other hand, there have been studies^6^,^8^ which have shown that weight and BMI have a negative correlation with heart rate recovery^8^ and resting heart rate^6^ in healthy adolescents. This inconsistency may be due to the fact that the participants in our study were athletes who used to eat healthily and spend long hours training every day.^20^ Moreover, Pepera et al^27^ study though have shown that the taller patient had a greater advantage when performing in a shuttle walking test but the same did not apply to healthy and active populations.

There are remarkable reasons why gender was not associated with cardiovascular function in this study, in contrast to the studies of healthy populations^8^. Firstly, one of the reasons is that the mechanism of the cardiovascular changes in athletes were similar despite the gender. Furthermore, the amount of men athletes is much more than the amount of the woman population (17%).

## Limitations- Future Suggestions

The current study has important strengths and limitations. The application of the above suggestions might be limited by various methodological issues. A larger sample would allow the generalisability of findings and the stratified analyses by sex, BMI, and sports practice time. Future studies should quantify the relationship between the maximum oxygen uptake, the cardiovascular parameters and smoking, in athletes. Also, it is possible to determine the correlating of the cardiovascular parameter' and maximum oxygen uptake' chanes in the different types of sports.

## Conclusion

The findings of the present study suggest that smoker athletes' physical fitness and cardiovascular health were lower than non-smokers, as means of higher heart rate recovery and recovery time. Athletes who smoke may have a higher risk of developing cardiovascular diseases during their life. Heart rate recovery and recovery time are proved a much more meaningful tool of assessing athlete's functional capacity and cardiovascular function for physical performance. Furthermore, the level of physical fitness and cardiovascular function of athletes depend on miscellaneous sports.

## References

[1] Liu H, Lee D, Jung S, Koo H, Kim E, Hwang S, Baek S, Jeon S, Kim J, Suh D (2014). Study design to Evaluate Association between smoking and intracranial atherosclerotic stenosis. Neurointervetion.

[2] Smoking leads to five- fold increase in heart disease and stroke in under- 50s.

[3] Messner B, Bernhard D (2014). Smoking and cardiovascular disease: mechanisms of endothelial dysfunction and early atherogenesis. Arteriosclerosis, Thrombosis, and Vascular Biology.

[4] Erdem A, Ayhan SS, Ozturk S, Ozlu MF, Alcelik A, Sahin S, Tosun M, Erdem FH, Gumustekin K, Yazici M (2012). Cardiac autonomic function in healthy young smokers. Toxicology and Industrial Health.

[5] Kim CS, Kim MK, Jung HY, Kim MJ (2017). Effects of Exercise Training Intensity on Cardiac Autonomic Regulation in Habitual Smokers. Ann Nonivasive Electrocardiology.

[6] Sellers E (2009). An Examination of Post Exercise Heart Rate and Recovery Time. Biology.

[7] De Borda A, Jost RT, Gass R, Nedel FB, Cardoso DM, Pohl H, Rechziegel MB, Corbellini VA, Paiva DN (2014). The influence of active and passive smoking on the cardiorespiratory fitness of adults. Multidisciplinary Respiratory Medicine.

[8] Hanifah R, Mohamed M, Zulkarnain J, Mohsein N, Jalaludin M, Majid H, Murray L, Cantwell M, Su T (2013). The Correlates of Body Composition with Heart Rate Recovery after Step Test: An Exploratory Study of Malaysian Adolescents. PLoS One.

[9] Kobayashi Y, Takeuchi T, Hosoi T, Loeppky J (2004). Effects of habitual smoking on cardiorespiratory responses to sub-maximal exercise. Journal of physiological anthropology and Applied Human Science.

[10] Erat M, Doğan M, Sunman H, Asarcıklı L D, Efe T H, Bilgin M, Çimen T, Akyel A, Yeter E (2016). Evaluation of heart rate recovery index in heavy smokers. Anatolian Journal of Cardiology.

[11] Sydó N, Merkely B, Gonzalez Carta KA, Becker D, Hussain N, Murphy J G, Sydó T, Lopez-Jimenez F, Allison T G (2018). Effect of Cardiorespiratory Fitness on Co-Morbidities and Mortality in Never, Past, and Current Smokers. The American Journal of Cardiology.

[12] Astrand P, Rodahl K, Dahl HA, Stromme SPO (2003). Textbook of work physiology. Physiological basis of exercise. Champagne Human Kinetics.

[13] Sydo N, Sydo T, Gonzalez Carta K A, Hussain N, Farooq S, Murphy J G, Merkely B, Lopez-Jimenez F, Allison T G (2018). Prognostic Performance of Heart Rate Recovery on an Exercise Test in a Primary Prevention Population. Journal of American Heart Association.

[14] Durmic T, Ðjelic M, Gavrilovic T, Antic M, Jeremic R, Vujovic A, Mihailovic Z, Zdravkovic M (2019). Usefulness of heart rate recovery parameters to monitor cardiovascular adaptation in elite athletes: The impact of the type of sport. Physiology International Journal.

[15] Buchheit M, Gindre C (2006). Cardiac parasympathetic regulation: respective associations with cardiorespiratory fitness and training load. American Journal Physiology-Heart and Circulation.

[16] Peretti-Watel P, Guagliardo V, Verger P, Pruvost J, Mignon P, Obadia Y (2003). Sporting activity and drug use: alcohol, cigarette and cannabis use among elite student athletes. Addiction.

[17] Papathanasiou G, Georgoudis G, Papandreou M, Spyropoulos P, Georgakopoulos D, Kalfakakous V, Evangelou A (2009). Reliability Measures of the Short International Physical Activity Questionnaire (IPAQ) in Greek Young Adults. Hellenic Journal of Cardiology.

[18] Pepera G, McAllister J, Sandercock G (2010). Long- term reliability of the Incremental Shuttle Walking Test in clinically stable cardiovascular disease patients. Physiotherapy.

[19] Pepera GK, Bromley PD, Gandercock GRH (2013). A pilot study to investigate the safety of exercise training and exercise testing in cardiac rehabilitation patients. British Journal of Cardiology.

[20] Jin-Jang D, Kim HC, Kim JK, Jung SY, Kim DY (2017). Effects of habitual smoking on cardiopulmonary function in taekwondo athletes. Journal of Exercise Rehabilitation.

[21] Papathanasiou G, Georgakopoulos D, Papa-georgiou E, Zerva E, Michalis L, Kalfakakou V, Evangelou A (2013). Effects of Smoking on Heart Rate at Rest and During Exercise, and on Heart Rate Recovery, in Young Adults. Hellenic Journal of Cardiology.

[22] Seiler S, Haugeni O, Kuffel E (2007). Autonomic Recovery after Exercise in Trained Athletes: Intensity and Duration Effects. Official Journal of the American College of Sports Medicine.

[23] Chen CL, Tang J S, Li PC, Chou PL (2015). Immediate Effects of Smoking on Cardiorespiratory Responses During Dynamic Exercise: Arm Vs. Leg Ergometry. Frontiers of Physiology.

[24] Gharbi Z, Dardouri W, Haj-Sassi R, Chamari K, Souissu N (2015). Aerobic and anaerobic determinants of repeated sprint ability in team sports athletes. Biology of Sport.

[25] Pepera GK, Sandercock GR, Sloan R, Cleland JJ, Ingle L, Clark AL (2012). Influence of step length on 6- minute walk test performance in patients with chronic heart failure. Physiotherapy.

[26] Pepera G, Ingle L, Sandercock GR (2015). Pre- dictors of the 6-minute walk test in patients with chronic heart failure. British Journal of Cardiac Nursing.

[27] Pepera G, Taylor MJD, Peristeropoulos A, Sandercock GRH (2013). Predictors of shuttle walking test performance in patients with cardiovascular disease. Physiotherapy.

[28] Sandercock GRH, Cardoso F, Almodhy M, Pepera G (2013). Cardiorespiratory fitness changes in patient receiving comprehensive outpatient cardiac rehabilitation in the United Kingdom: a multicentre study. Heart.

[29] Cardoso FM, Almodhy M, Pepera G, Stasinopoulos DM, Sandercock GR (2017). Reference values for the incremental shuttle walk test in patients with cardiovascular disease entering exercise-based cardiac rehabilitation. Journal of Sports Sciences.

